# A novel baiting microcosm approach used to identify the bacterial community associated with *Penicillium bilaii* hyphae in soil

**DOI:** 10.1371/journal.pone.0187116

**Published:** 2017-10-27

**Authors:** Behnoushsadat Ghodsalavi, Nanna Bygvraa Svenningsen, Xiuli Hao, Stefan Olsson, Mette Haubjerg Nicolaisen, Waleed Abu Al-Soud, Søren J. Sørensen, Ole Nybroe

**Affiliations:** 1 Section for Microbial Ecology and Biotechnology, Department of Plant and Environmental Sciences, University of Copenhagen, Frederiksberg C, Denmark; 2 State Key Laboratory of Ecological Pest Control for Fujian and Taiwan Crops, Fujian Agriculture and Forestry University, Fuzhou, China; 3 Section of Microbiology, Department of Biology, University of Copenhagen, Copenhagen, Denmark; Friedrich Schiller University, GERMANY

## Abstract

It is important to identify and recover bacteria associating with fungi under natural soil conditions to enable eco-physiological studies, and to facilitate the use of bacterial-fungal consortia in environmental biotechnology. We have developed a novel type of baiting microcosm, where fungal hyphae interact with bacteria under close-to-natural soil conditions; an advantage compared to model systems that determine fungal influences on bacterial communities in laboratory media. In the current approach, the hyphae are placed on a solid support, which enables the recovery of hyphae with associated bacteria in contrast to model systems that compare bulk soil and mycosphere soil. We used the baiting microcosm approach to determine, for the first time, the composition of the bacterial community associating in the soil with hyphae of the phosphate-solubilizer, *Penicillium bilaii*. By applying a cultivation-independent 16S rRNA gene-targeted amplicon sequencing approach, we found a hypha-associated bacterial community with low diversity compared to the bulk soil community and exhibiting massive dominance of *Burkholderia* OTUs. *Burkholderia* is known be abundant in soil environments affected by fungi, but the discovery of this massive dominance among bacteria firmly associating with hyphae in soil is novel and made possible by the current bait approach.

## Introduction

Soil is a highly complex environment and, as a consequence of its many microhabitats, soil harbors the highest known bacterial diversity on earth [1, 2]. However, soil fungi account for the predominant fraction of the soil microbial biomass [3] and the total length of fungal hyphae in soil can exceed 1000 m per g [4]. Hence, fungal hyphae provide a large surface for interactions with other soil microorganisms, and these microhabitats are referred to as bacterial-fungal interfaces [5, 6, 7]. Indeed, the fungal hyphae also represent efficient dispersal networks for their associated bacteria [8].It is becoming increasingly clear that fungi affect both the size and composition of bacterial communities in their surroundings. A number of studies have compared bulk soil with and without growth of hyphae. These studies may not be able to distinguish between bacteria closely associated with hyphae and bacteria affected by hyphae from some distance. Nevertheless this approach has, for example, revealed that the arbuscular mycorrhizal fungus *Glomus hoi* results in increased abundance of Firmicutes [9], while the saprotrophic fungus *Lyophyllum* sp. strain Karsten increases the abundance of *Burkholderia* and *Pseudomonas* [10]. Another approach has been to isolate bacteria from the mycosphere, *i*.*e*. bacteria associated with mycelial bundles in soil [11]. A recent comparative study revealed a core mycosphere bacterial community, including representatives of the families *Acetobacteraceae*, *Chrhoniobacteraceae*, *Planctomycetaceae*, *Conexibacteraceae and Burkholderiaceae*, but at the same time showed clear differences between mycosphere communities from different mushroom-forming fungi [12]; differences that may be caused by different composition of hyphal exudates [13, 14].

Bacteria that associate tightly with hyphae may provide important services to their fungal host. This is exemplified by mycorrhizal helper bacteria that assist mycorrhiza formation or promote the function of the mycorrhizal symbiosis [15]. However, analysis of hypha-associated bacterial communities is experimentally challenging [16]. Different laboratory model systems have been developed, which allow colonization of fungal cultures by extracted soil bacterial communities, or by introduced isolates under controlled conditions [13, 16, 17, 18]. These studies have documented the presence of specific hypha-associated genera (*Bacillus*) or broader groups (Oxalobacteria, *Alphaproteobacteria*, *Gammaproteobacteria*), depending on the fungus and the taxonomic resolution of the studies. However, we still need to develop approaches enabling in-depths molecular identification of the bacterial communities that associate with, in particular hyphae of non-mycorrhizal fungi, under more natural soil conditions.

The first objective of the current study was, therefore, to establish a novel baiting method to recover bacteria associating with fungal hyphae in a close to natural soil system. We used the phosphate (P) solubilizing fungus *Penicillium bilaii* as bait in the system. The next objective was to characterize the hypha-associated microbiome of *P*. *bilaii* comprehensively by 16S rRNA gene-targeted amplicon sequencing.

## Materials and methods

### Experimental set-up

**Fig 1:**
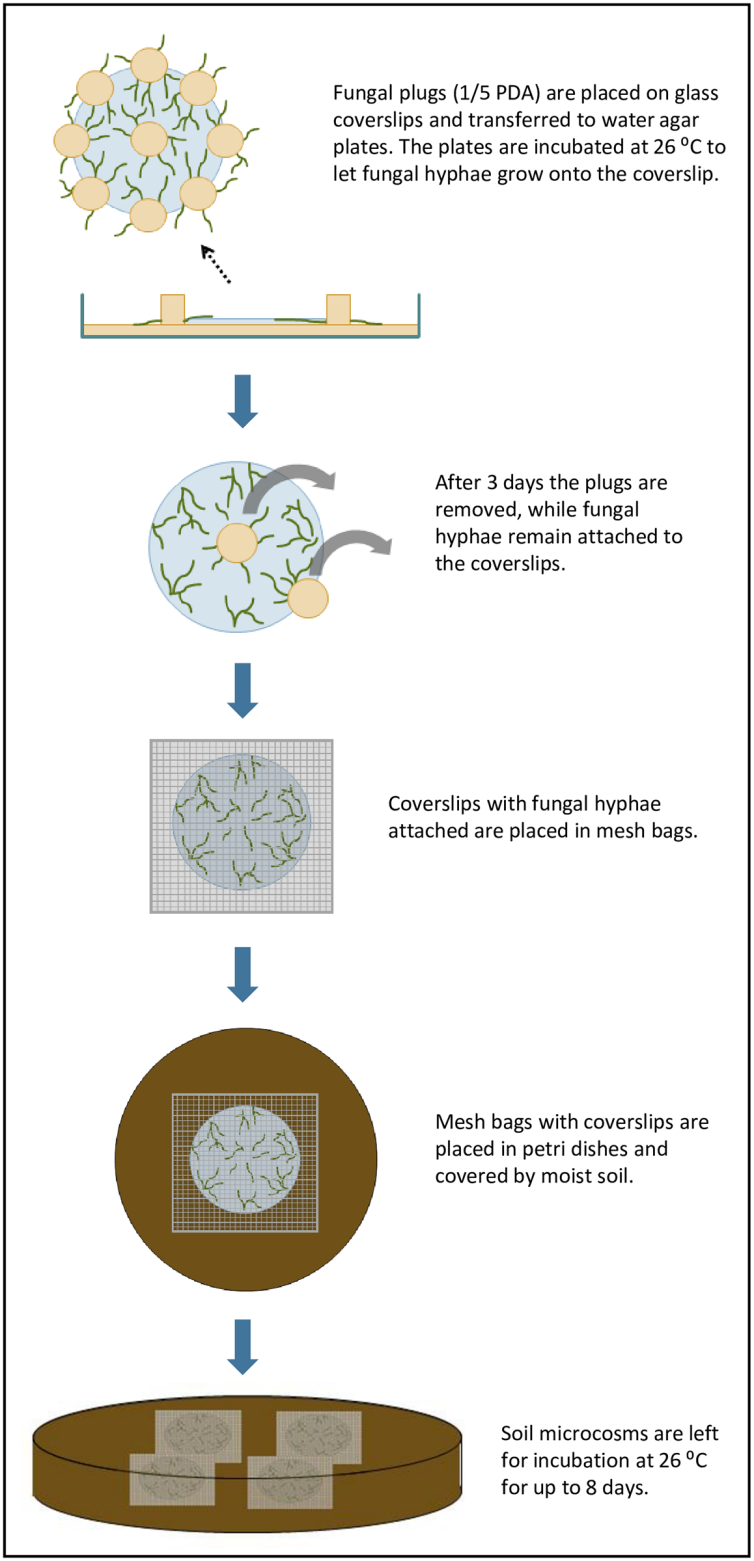
Schematic diagram showing the microcosm setup for isolation of hypha-associated bacteria from soil.

We developed a model system to recover bacteria that associate with the hyphae of *P*. *bilaii* under close-to-natural soil conditions (Fig 1). The fungal hyphae grew from agar plugs and established on glass cover slips serving as a solid support. After removal of the plug, the cover slips were subsequently transferred to mesh bags and buried in soil. Hyphal growth and metabolic activity was quantified by Calcofluor White and FUN1 staining, respectively, before cover slips were transferred to soil microcosms. Samples were taken after different times in the soil to determine the presence and activity of the hyphae, and to recover hypha-colonizing bacteria.

### Set-up of soil microcosm systems

Spore suspensions of *P*. *bilaii* strain ATCC 20851 were preserved as aliquots in 10% glycerol at -80°C until use. The fungus was routinely grown on 1/5 strength Potato Dextrose Agar (PDA; Difco Laboratories). To obtain hyphal material for transfer to soil microcosms, *P*. *bilaii* was incubated on 1/5 strength PDA overnight at 26°C. Fungal plugs were subsequently placed on sterilized cover slips (diameter = 25 mm), which were then transferred to 1.5% water agar plates and incubated for three days at 26°C. At this time, the fungal plugs were carefully removed from the cover slip using a scalpel to avoid destroying hyphal extensions. The cover slips were then transferred to sterile mesh bags (one cover slip per mesh bag) made of Polyamide (Sintab Produkt AB, fabric 6111–005043, mesh width 50 μm), which were subsequently sealed by heating. Mesh bags were transferred to soil microcosms so that each microcosm contained four mesh bags that were all covered by soil, hence ensuring good contact between the mesh bag and the soil. Slides without fungal hyphae were processed in parallel as controls.

Soil microcosms consisted of Petri dishes containing 100 g of sandy loam soil (clay 16.4%, silt 17.3%, fine sand 33.3%, coarse sand 31.2%, organic matter 1.7%, pH 6.4) obtained from a long-term nutrient depletion trial at the University of Copenhagen experimental farms [19]. The soil was unfertilized from 1964 to 1995, and from 1996 onwards it was fertilized with 60 kg mineral fertilizer N ha^-1^ year^-1^. The soil did not receive P or K fertilizers and was mainly cropped with cereals. Microcosms were maintained at a soil water content of 14% w/w and were incubated for up to 8 days at 26°C.

### Microscopic observations

Presence and viability of *P*. *bilaii* hyphae were determined by the LIVE/DEAD^®^ Yeast Viability Kit (Thermo Fisher Scientific) according to manufacturer’s instructions. The kit contains Calcofluor^®^ White M2R, which labels cell-wall chitin with blue-fluorescence regardless of metabolic state [20]; and the FUN^®^ 1 two-color fluorescent viability probe. Plasma membrane integrity and metabolic function of fungi are required to convert the yellow-green-fluorescent intracellular staining of FUN^®^ 1 into red-orange staining (https://www.thermofisher.com/order/catalog/product/L7009). Briefly, coverslips with fungal hyphae were recovered from 0, 4, 6 and 8-days old soil microcosms, respectively. At least two coverslips were used for either FUN 1 or Calcofluor White M2R staining, respectively. Fungal hyphae stained with 10 μM FUN 1 were incubated at 30°C for 1 h, while hyphae stained with 25 μM Calcofluor White M2R were incubated in the dark at room temperature for 15 min. A negative (killed) control for hyphal viability was set as follows: after removing fungal plugs, fungal hyphae on coverslips were left at room temperature for 5 days without any nutrients to let the hyphae die naturally. Hyphae stained with FUN 1 or Calcofluor White were observed with a Zeiss Axioplan 2 epifluorescence/light microscope equipped with FTIR or DAPI filter sets, and a 63× oil objective lens (Carl Zeiss; total magnification was 630 x). Images were taken with a Zeiss AxioCam digital camera, viewed on Zeiss AxioVision image analysis software, and processed with the ImageJ program.

Hyphae-colonizing bacteria on the coverslips were visualized by staining with a SYBR Green (Life Technologies) stock solution diluted 1:100 for 5 minutes. Fluorescence microscopy was made by a Nikon Ellipse TE300 inverted microscope equipped with filters for blue excitation and green emission, and operated at 100 or 400 times magnification. Images were taken with a Nikon digital camera, model D5200.

### Recovery of hypha-associated bacteria

To recover *P*. *bilaii* hyphae and their associated bacteria, colonized coverslips were washed gently twice with 500 μl of sterile Milli Q-purified water (Millipore) after incubation in the soil for 8 days. Subsequently, 200 μl of Milli Q water were added to the surface of the slide, and fungal hyphae and associated bacteria were scraped off by a scalpel. Non-colonized cover slips without fungal hyphae were treated as above. These suspensions were used for quantification of bacteria by spread plating, and for DNA extraction.

### Quantification of bacteria recovered from colonized versus non-colonized coverslips

Bacteria were quantified separately in suspensions containing *P*. *bilaii* hyphae and hypha-associated bacteria, or in suspensions obtained from cover slips that were not colonized by *P*. *bilaii*. The suspensions were vortexed before serial dilutions were made and spread on 1/10 strength Reasoners medium 2 agar (R2A; Difco Laboratories) supplemented with 50 μg/ml Nystatin (Sigma-Aldrich) to prevent fungal growth. Plates were incubated at 21°C for 48h before colonies were enumerated.

### Microcosm validation

**Fig 2:**
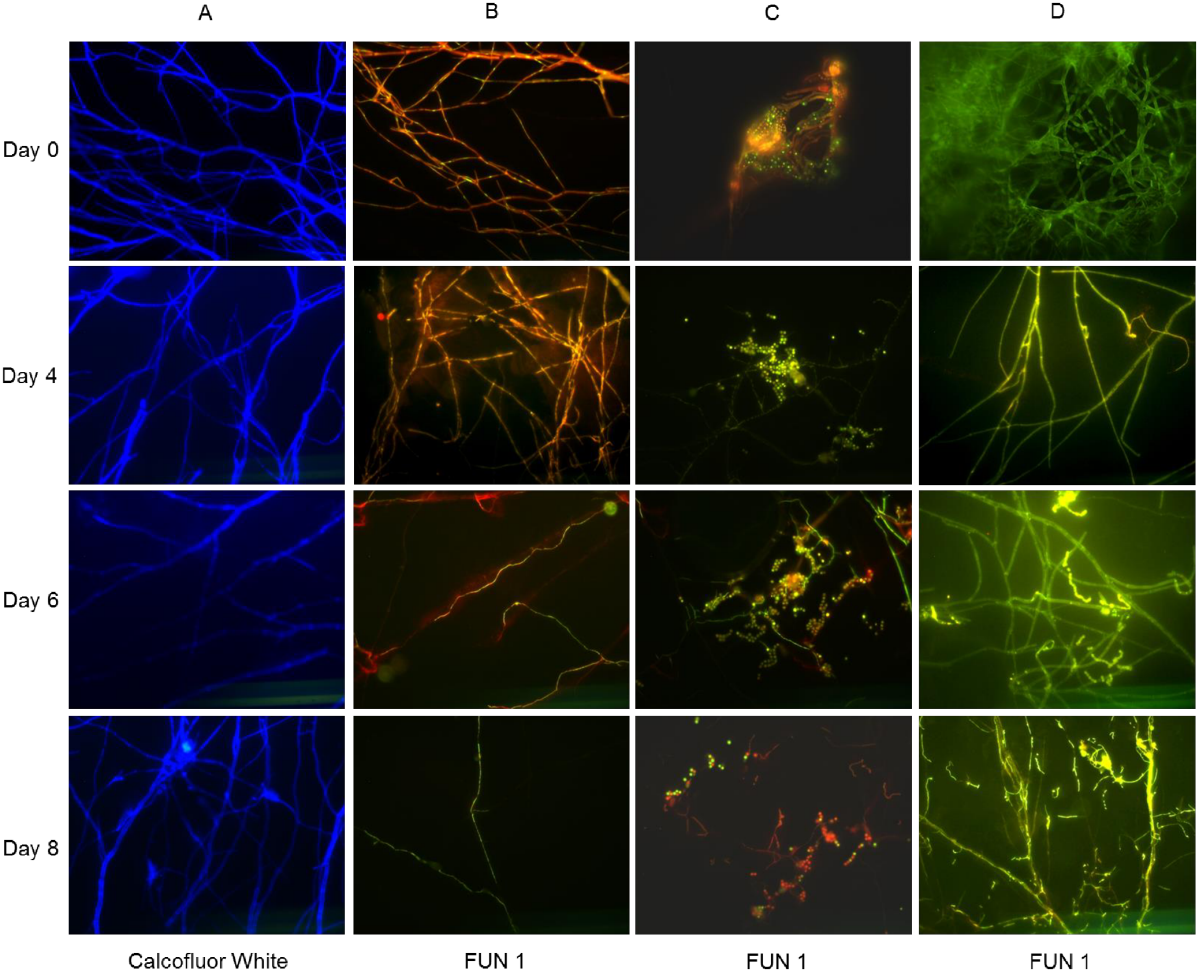
Visualization of *P*. *bilaii* hyphal growth and hyphal viability. Glass cover slips were recovered from soil microcosms at day 0, 4, 6 and 8. Column A: Calcofluor White M2R was used to stain the cell walls of *P*. *bilaii*. Columns B, C and D: FUN 1 stain was used to determine metabolically active (B) or inactive (D) hyphae, as well as active and inactive spores (C). Killed hyphae were set as a negative control (Column D, Day 0). All images were obtained at 630 times magnification.

Before cover slips were transferred to soil microcosms, hyphal growth onto the cover slips was confirmed by fluorescence microscopy after Calcofluor White staining of hyphal cell walls (Fig 2, Column A, Day 0). Furthermore, staining of the hyphae with FUN ^®^1 showed that red-orange structures were formed in hyphae, which is characteristic of metabolically active hyphae (Fig 2, Column B, Day 0), while killed hyphae showed diffuse bright green fluorescence (Fig 2, Column D, Day 0). Furthermore, fungal spores showed either red-orange or green fluorescence indicating a mixed population of active and inactive spores at this stage (Fig 2, Column C, Day 0).

Bacteria in bacterial suspensions obtained from cover slips colonized by *P*. *bilaii* and from non-colonized control slips were enumerated by CFU counts on day 8. From colonized cover slips we obtained 560 ± 72 CFU per coverslip (n = 3) while from non-colonized controls we obtained 5±2 CFU per coverslip (n = 3). This result supports the notion that the overwhelming majority of bacteria isolated from colonized cover slips were indeed hypha-associated and not bacteria that attached to the cover slip.

### DNA extraction

DNA was extracted from duplicate, 0.5 g soil samples by the Fast DNA Spin Kit for Soil (MP Biomedicals) according to the manufacturer’s protocol. DNA from the suspensions of *P*. *bilaii* hyphae and hypha-associated bacteria was extracted with the DNeasy Blood & Tissue kit (QIAGEN) following the manufacturer’s protocol for DNA extraction from bacteria including pretreatment for Gram-positive bacteria. Extractions were made for duplicate samples of hyphae and hypha-associated bacterial suspensions, each containing material from 8 colonized cover slips. Pooling was required due to the low bacterial numbers on individual slips. Prior to extraction, samples were centrifuged for 5 min at 10,000 x g, and DNA was extracted from the pellet plus 100 μl of the supernatant. Additional supernatant was discarded.

### 16S rRNA gene high throughput amplicon sequencing

A 466 bp fragment of the variable V3 and V4 regions of the 16S rRNA gene was amplified by PCR using the primer pair 341F (5’-CCTAYGGGRBGCASCAG-3’) and 806R (5’-GACTACNNGGGTATCTAAT-3’) [21]. The PCR reactions were set up in 50 μl containing 2.5 U Taq DNA polymerase (Sigma-Aldrich), 1x PCR Buffer (Sigma-Aldrich), 200 μM of each dNTP and 0.5 μM of each primer. Thermal cycling conditions were following: 94°C for 10 min, followed by 35 cycles of 94°C for 30 s, 56°C for 1 min, 72°C for 1 min and a final extension at 72°C for 7 min. PCR products were purified from a 1% agarose gel using a Gel Extraction kit (QIAGEN), and DNA concentrations were quantified on a Qubit 2.0 fluorometer (Invitrogen) with the Qubit dsDNA HS Assay Kit (Invitrogen). A second PCR step was performed to add adapters and indexes to the amplified 16S rRNA gene fragments. Each of the fusion primers had a barcode sequence of eight nucleotides that was used to identify each sample. The PCR mix (20 μL) contained: 1X AccuPrime^™^ PCR Buffer II (1.5 mM MgCI_2_), 0.24 U AccuPrime^™^ Taq DNA Polymerase (Life Technologies), 0.5 μM of each primer, 2 μL diluted template (Life Technologies, Carlsbad, CA). The PCR incubation conditions were: An initial hot-start step incubation at 94°C for 2 min, followed by 15 cycles at 94°C for 20s, 56°C for 20s and 68°C for 30s, and final extension at 68°C for 5 min. The samples were incubated at 70°C for 3 min. Long amplicons were purified by Agencourt AMpure XP (Beckman Coulter). Subsequently, the purified PCR products were quantified using Qubit dsDNA Assay kit as described above and then pooled in one tube by adding equimolar concentration of each sample. The pooled samples were concentrated using the DNA clean and concentrator-5 kit (Zymo Research, Orange, CA) and sequenced on Illumina MiSeq V2 kit (250 paired ends, Illumina, San Diego, CA) following the manufacturer’s instructions.

Sequence analysis was performed using the CLC Genomics Workbench with the Microbial Genomics Module (QIAGEN). Merged paired end reads were trimmed using the software’s default settings with a quality score limit of 0.01 (Phred score 20) resulting in sequences of 401 bp. The partial 16S rRNA gene sequences were clustered and assigned to operational taxonomic units (OTUs) with 97% similarity using the SILVA 16S database version 119. During the clustering step, chimeric sequences were removed using the software’s default settings. Phylogenetic Neighbor Joining trees of bacterial taxa from hyphal communities affiliated with the genera *Burkholderia*, *Pseudomonas* and *Massilia* were constructed from aligned sequences using Maximum Likelihood Phylogeny with 1000 bootstrap replicates. Reference sequences were downloaded from GenBank and trimmed to the same length as hypha-associated sequences, thus resulting in alignments of 375–400 unambiguously aligned nucleotides. Alignments, rarefaction analyses and tree construction were done with the CLC software. Diversity indices were calculated on OTU level using the PAST software version 2.17 [22].

## Results

### Hyphal growth, viability and colonization by bacteria in soil

**Fig 3:**
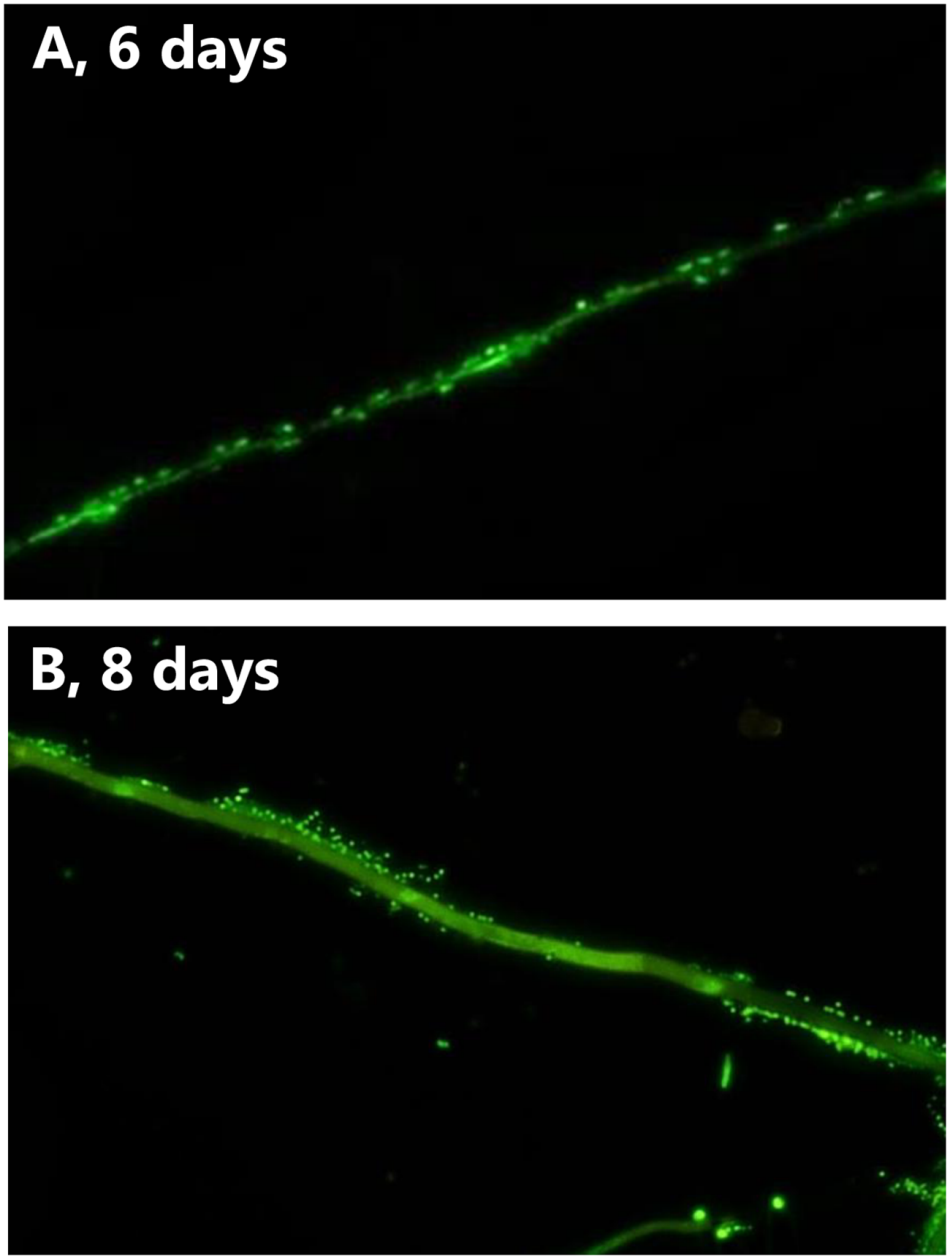
Colonization of *Penicillium bilaii* hyphae by bacteria after incubation for A: 6 days and B: 8 days in soil. Hyphae and bacteria were stained by SYBR Green and the microscopy image was obtained at 400 times magnification.

The cover slips colonized by *P*. *bilaii* hyphae were transferred to soil and analyzed at different time points up to 8 days after the transfer. Intact hyphae were seen on the coverslips throughout the experiment (Fig 2, Column A, Day 4–8), and both metabolically active and inactive hyphae were observed at days 4, 6 and 8 (Active: Fig 2, Column B, Day 4–8, Inactive: Column D, Day 4–8). Interestingly, *P*. *bilaii* spore activity seemed to increase after 4 days in the soil (Fig 2, Column C, day 4–8) and after 8 days we observed growth of novel, metabolically active hyphae from germinating spores (Fig 2, Column C, Day 8). Attachment of bacteria to the hyphae was observed after incubation for 6 to 8 days in the soil (Fig 3).

### Culture independent analysis of soil and hypha-associated bacterial communities

High throughput amplicon sequencing of the V3-V4 regions of the 16S rRNA genes from microcosm soil and hypha- associated bacteria resulted in the range of 10,756 to 47,271 sequences after quality filtering and chimera removal. The sequences clustered into 2863 and 590 OTUs at the 97% sequence similarity level for soil and hyphal samples, respectively. Rarefaction analyses for non-rarified data supported that our sequencing depth covered the majority of the hypha-associated bacterial community, while this was not the case for the bulk soil community (S1 Fig).

**Fig 4:**
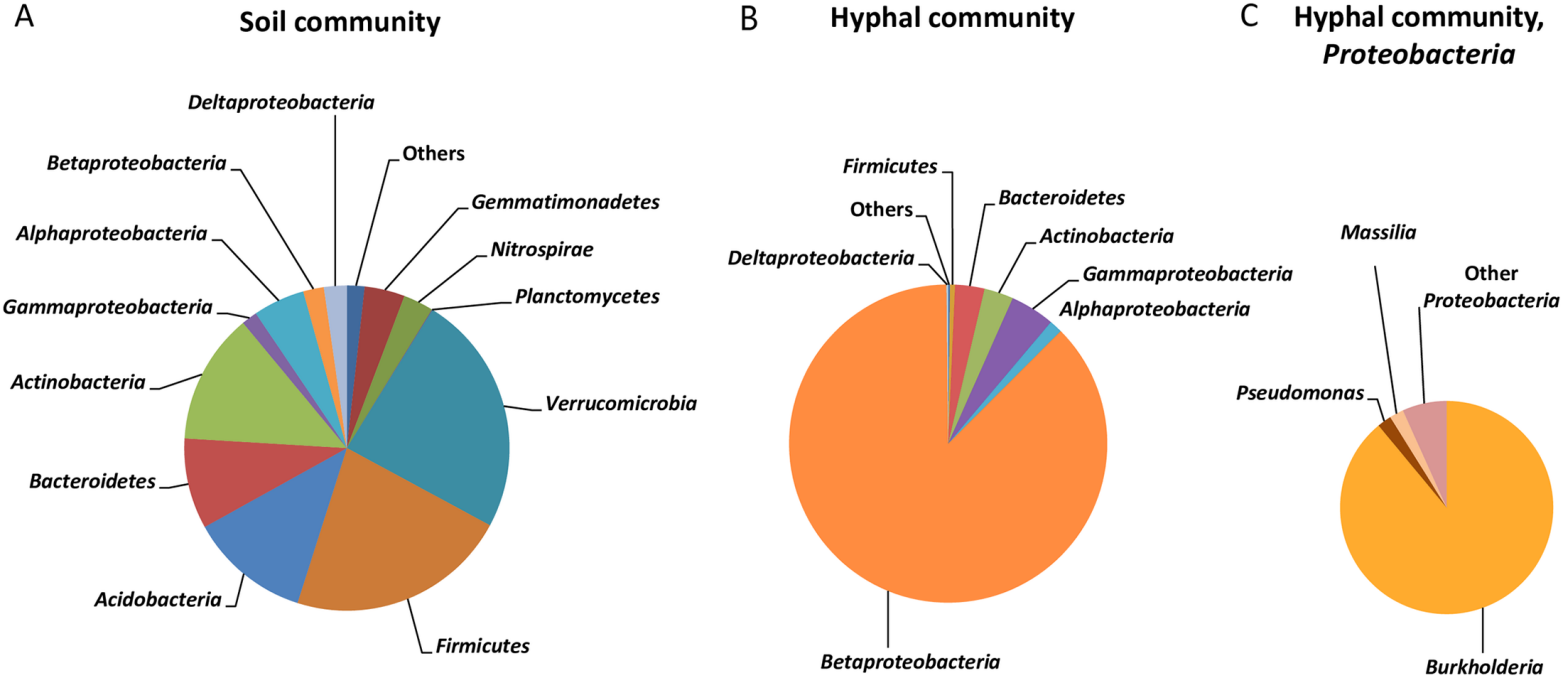
Composition of microbial communities in A: Bulk soil or B and C: Attached to the hyphae of *P*. *bilaii*. Relative abundances of taxa are shown on phylum level in panel A and B (*Proteobacteria* are displayed at the class level), while panel C depicts the distribution of proteobacterial genera from the hyphae-associated community. “Others” represent taxa with < 1% relative abundance. Data for either microbial community is obtained from two replicates.

In soil the most abundant phyla were *Verrucomicrobia* and *Firmicute*s, each accounting for 22–24% of the sequences. A*ctinobacteria*, *Acidobacteria*, *Proteobacteria* and *Bacteroidetes* were each represented by 9–12% of the sequences, *Gemmatimonadetes* and *Nitrospirae* by 3–4%, while other phyla, each represented by less than 1% of the sequences accounted for 2% of the sequences (Fig 4A).

The fungal hyphae selected a distinct part of the soil bacterial community, as only four phyla found in soil were represented by more than 0.5% of the sequences obtained from hypha-associated bacteria (Fig 4B). The hypha-associated bacterial community was noticeably dominated by *Proteobacteria*, accounting for 93% of the sequences, and in particular by *Betaproteobacteria*. Hence, *Proteobacteria* had a significantly higher relative abundance in the hypha-associated community than in the soil community (P = 0.0023, Tukey test). *Bacteroidetes*, *Actinobacteria* and *Firmicutes* were represented by 0.5–3% of the sequences, while other phyla accounted for 0.2% of the sequences. The strong selection for a distinct bacterial community by the fungal hyphae was further supported by a lower diversity, estimated by the Shannon H index. The value for the soil community was 6.40 ±0.13 while the value for the hyphal community was significantly lower (1.65±0.04, P = 0.00078, Tukey test). Further, the hypha-associated community displayed a significantly lower richness than the soil community as the Chao1 index for the soil community was 2070±508 as compared to 384±49 for the hypha-associated community (P = 0.043, Tukey test).

As the hypha-associated bacterial community was dominated by *Proteobacteria*, we undertook a further phylogenetic analysis at the genus level for this group. Interestingly, the majority of proteobacterial sequences (89%) were assigned to *Burkholderia* (Fig 4C). *Pseudomonas* and *Massilia* were the second most abundant genera; however each of these genera only accounted for 2% of the proteobacterial sequences. Other genera, each accounting for less than 1% of all the sequences, represented 7% of the sequences. For comparison, the relative abundance of proteobacterial sequences from bulk soil that could be assigned to *Burkholderia*, *Pseudomonas* or *Massilia* was < 1% for all three genera. Furthermore, we performed 16S rRNA gene sequencing on DNA recovered from pure suspensions of *P*. *bilaii* spores but did not recover any *Burkholderia* related sequences.

**Fig 5:**
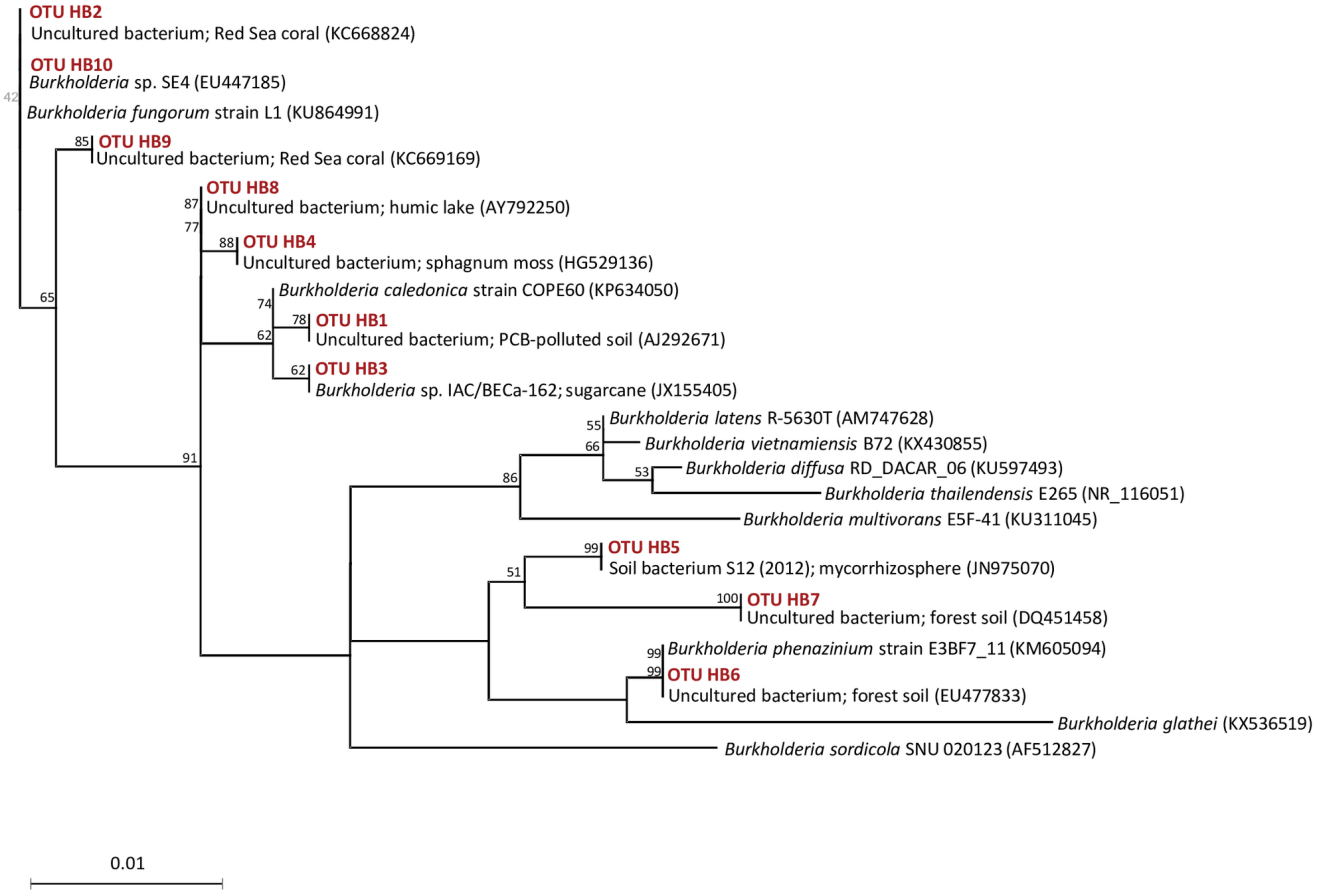
Neighbor-joining tree of bacteria associated with *P*. *bilaii* hyphae belonging to the genus *Burkholderia*. The tree was constructed from 375 unambiguously aligned nucleotides from the V3-V4 regions of the 16S rRNA gene. Sequences derived from hyphae-associated bacteria from the microcosms are designated OTU HB. Bootstrap values are based on 1000 pseudo-replications, and only values > 50% are shown.

Phylogenetic neighbor joining analysis showed that, despite the dominance of *Burkholderia* among the hypha-associated bacteria, sequences affiliated with *Burkholderia* were only represented by 10 different OTUs based on 97% similarity. These OTUs along with their closest published relatives, included several sequences from soil or mycorhizosphere, but even from corals and from aquatic samples (Fig 5). The phylogenetic analysis of sequences representing *Pseudomonas* and *Massilia* shows more diverse populations with closest relatives derived from a wide range of different environments (S2 and S3 Figs).

## Discussion

It is important to identify and recover bacteria associating with fungi under natural soil conditions to enable studies of both partners’ eco-physiology, and to facilitate the use of bacterial-fungal consortia in environmental biotechnology. Consequently, we have developed a novel type of baiting microcosm, in which fungal hyphae and bacteria interact directly in the soil. Controlled, laboratory-based microcosms have recently been developed, in which fungal hyphae grow out onto, or into, nutrient-poor media and are confronted with bacteria extracted from soil or rhizosphere [16, 18, 17]. Compared to the current system, these microcosms suffer from the bias arising from extraction of soil or rhizosphere bacteria and from the potential difference in composition of hyphal exudates produced in culture and in real soil conditions. A few systems have been designed, in which bacteria and fungi actually encounter each other in soil but these systems have so far exclusively been used to obtain bacterial isolates migrating along hyphal highways extending out of the soil and reaching a target cultivation medium, where the bacteria can be recovered [8, 23].

For the current system, hyphal growth is established on a solid support placed in the soil. This enables the recovery of hyphae, and bacteria that attach to the hyphae after two washing steps. It hence minimizes the abundance of bacteria that are affected by the fungal hyphae while being located at some distance in the soil. Several previous studies have relied on comparison of soil with or without (arbuscular mycorrhiza) fungal hyphae [9, 24]. Other studies have characterized bacterial communities in the mycosphere [12], where bacteria have a less defined association with the hyphae. The current baiting system offers the advantage of recovering bacteria that associate with fungal hyphae and can be applied to several fungi living in close to natural habitats. *P*. *bilaii* was disconnected from its original food base (the 1/5 PDA plug) at the transfer to soil, but never the less maintained a population of active hyphae throughout the experiment. Inactive hyphae were however present at all times, and with time, active hyphae were entangled with inactive ones making it difficult to assess the dominant metabolic state (active or inactive). Importantly, the hyphae did not show signs of degradation. It has previously been shown that bacteria attach differentially to viable active (vital) versus inactive (non-vital) hyphae of arbuscular mycorrhizal fungi [14]. For the current study we may have recovered bacteria that attach to both active and inactive *P*. *bilaii* hyphae during the incubation in soil. We propose the current method be used to identify bacteria associating with active, growing hyphae as well as inactive, decaying hyphae in soil.

The current study determined for the first time the composition of the bacterial community associating with hyphae of the saprophytic phosphate-solubilizing biofertilizer *P*. *bilaii* in natural soil. We found a hypha-associated bacterial community with a very low diversity as compared to the bulk soil community by cultivation-independent 16S rRNA gene-targeted high throughput amplicon sequencing. The low diversity is in agreement with previous reports of fungal-affected soil as compared to bulk soil [10, 12]. However, the current decrease in diversity is even more pronounced. This is probably due to the narrow focus on bacteria associated with hyphae rather than on bacteria generally affected by fungi, which is made possible by the current soil microcosm bait approach.

We document a very considerable dominance of *Burkholderia* OTUs among bacteria closely associated with fungal hyphae in soil, but also an enrichment (or selection) of *Pseudomonas* and *Massilia* OTUs. The latter two genera have both been found in association with other fungi or in soil affected by growing fungal hyphae [10, 16], and in particular *Burkholderia* co-occurs with fungi. By cultivation-dependent approaches *Burkholderia* has been found in *Pinus sylvestris-Suillus bovinus* mycorhizosphere [25], in soil associated with growing *Lyophyllum* sp. hyphae [10], and associated with hyphae of the fungi *Trichoderma harzianum*, *Mucor hiemalis* and *Rhizoctonia solani* [17, 26]. In addition, molecular studies have recently shown co-occurrence of fungal and *Burkholderia* OTUs in 266 soil samples [27], and that *Burkholderia* is part of the mycosphere core microbiome for a panel of 24 mushroom-forming fungi [12]. However, as recently stated by Stopnisek et al. [27], the extent and specificity of the fungal-*Burkholderia* associations in soil are still not fully understood. The current study suggests that *Burkholderia* bacteria colonize *P*. *bilaii* hyphae in soil and then establish in close association with the hyphae. Such a life style is compatible with observations of chemotaxis towards, and adherence of selected *Burkholderia* strains to fungal surfaces under laboratory conditions [27, 28]. *Burkholderia* associated with hyphae may originate from fungal spores, either internally or borne on the spore surfaces [29, 30]. However, we did not recover *Burkholderia* related sequences from spore suspensions, and hence conclude that these bacteria were recruited from the soil environment. The composition of hyphal exudates is probably the key for shaping hypha-associated bacterial communities [13]. Exudate composition of *P*. *bilaii* has been extensively characterized because of the fungal ability to solubilize phosphate. *P*. *bilaii* decreases the pH of both buffered and non-buffered growth media and releases organic acids primarily citric, malic, and oxalic acid [31]. The conditions at the hyphal surface that could be predicted from these (laboratory) experiments seem to favor *Burkholderia*, as these bacteria prefer acid growth conditions [32]; and the ability to grow on oxalate, citrate and malate is widespread in at least so-called plant beneficial *Burkholderia* [33]. Furthermore, oxalotrophs belonging to the families *Oxalobacteriaceae* and *Pseudomonaceae* are often found in association with fungi [10, 16, 34] as exemplified here by the genera *Massilia* and *Pseudomonas*.

Half of the *Burkholderia* OTUs that were recovered from hyphae clustered with representatives of these plant beneficial species *(B*. *caledonica*, *B*. *fungorum*, *B*. *phenazinium*), while OTUs clustering with the *B*. *cepacia* complex or with plant pathogenic *Burkholderia* species were not encountered. Interestingly, the current *Burkholderia*, *Massilia* and especially *Pseudomonas* OTUs often group together with sequences obtained from animal epithelium or faeces. We speculate that this may reflect the similarity between fungal and animal innate immune systems used to select specific bacterial communities to cover their surfaces as discussed by Ipcho et al. [35]. However, the results of the current phylogenetic alignment come from analysis of relatively short 16S rRNA gene sequences, and should be interpreted with caution.

During recent years, the functionality of bacterial communities interacting with fungal hyphae has received increasing attention. For example, hypha-associated *Burkholderia* have been recognized as a source of fungal antagonists able to control the pathogen *Rhizoctonia* causing sheath blight in rice [26]. However, the same study reported on *Burkholderia* able to promote fungal growth. On the same lines, bacteria associating with the hyphae of mycorrhizal fungi include so-called helper bacteria that promote mycelial growth and mycorrhiza formation [15, 16]. We speculate that such beneficial bacteria may even be associated with the hyphae of other fungi, including *P*. *bilaii* and are currently investigating a panel of isolates cultivated from *P*. *bilaii* hyphae for their potential as helper bacteria.

## Supporting information

S1 Fig Rarefaction curves. Curves for the number of observed OTUs (>97% similarity) in samples from hyphae-associated bacteria (yellow and orange) and from soil (blue and purple).
(TIFF)

S2 Fig Neighbor-joining trees of bacteria associated with *P*. *bilaii* hyphae belonging to the genus *Pseudomonas*. The trees were constructed from 375–400 unambiguously aligned nucleotides from the V3-V4 regions of the 16S rRNA gene. Sequences derived from hyphae-associated bacteria from the microcosms are designated OTU HP/HM. Bootstrap values are based on 1000 pseudoreplications, and only values > 50% are shown.
(TIFF)

S3 Fig Neighbor-joining trees of bacteria associated with *P*. *bilaii* hyphae belonging to the genus *Massilia*. The trees were constructed from 375–400 unambiguously aligned nucleotides from the V3-V4 regions of the 16S rRNA gene. Sequences derived from hyphae-associated bacteria from the microcosms are designated OTU HP/HM. Bootstrap values are based on 1000 pseudoreplications, and only values > 50% are shown.
(TIFF)
