# Supplementary figures and images for: A novel baiting microcosm approach used to identify the bacterial community associated with Penicillium bilaii hyphae in soil
Source: PLoS One. 2017 Oct 27;12(10):e0187116. doi: 10.1371/journal.pone.0187116 (PMC5659649; doi:10.1371/journal.pone.0187116)

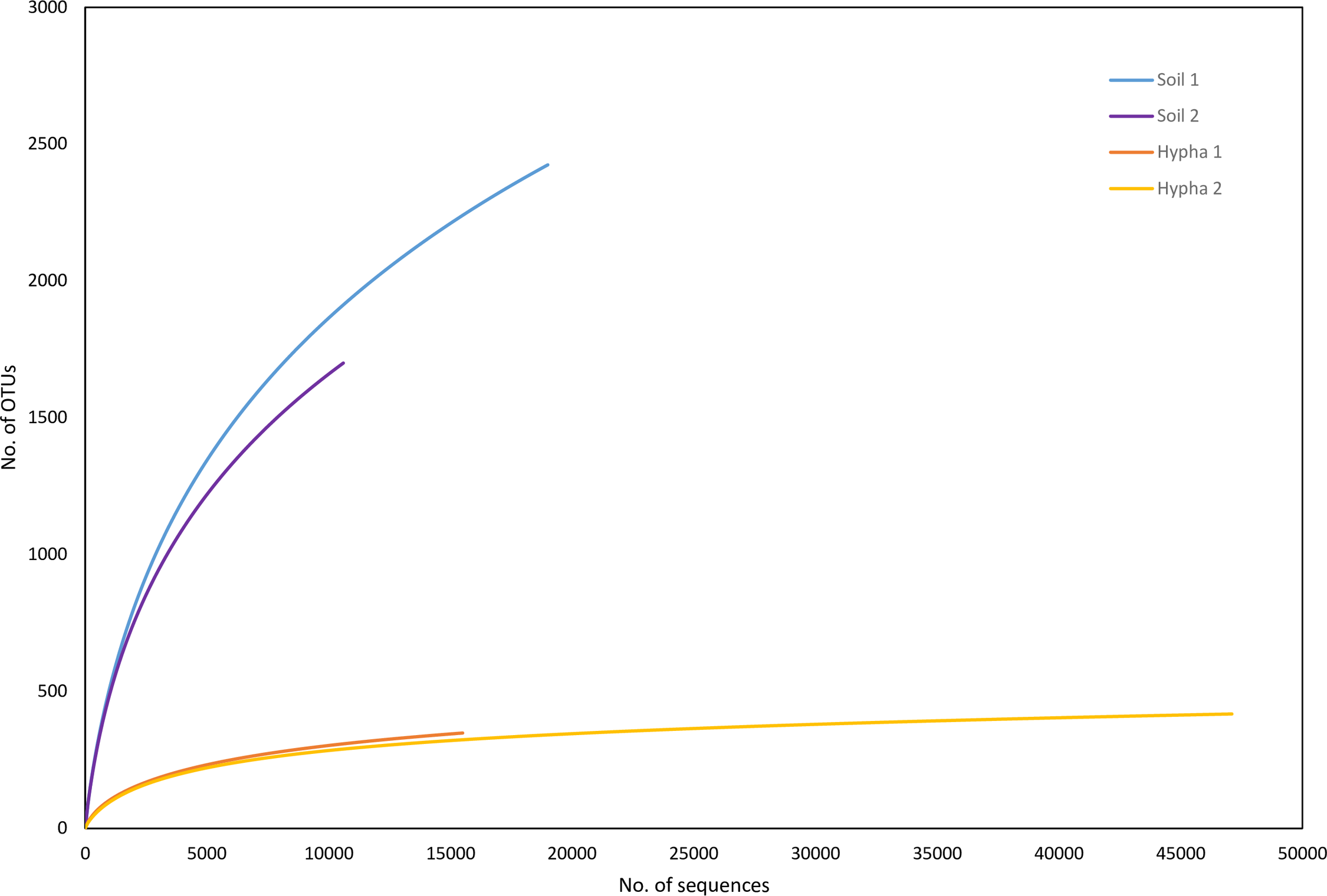

Supplement: S1 Fig — Curves for the number of observed OTUs (>97% similarity) in samples from hyphae-associated bacteria (yellow and orange) and from soil (blue and purple). (TIFF) [file pone.0187116.s001.tiff]

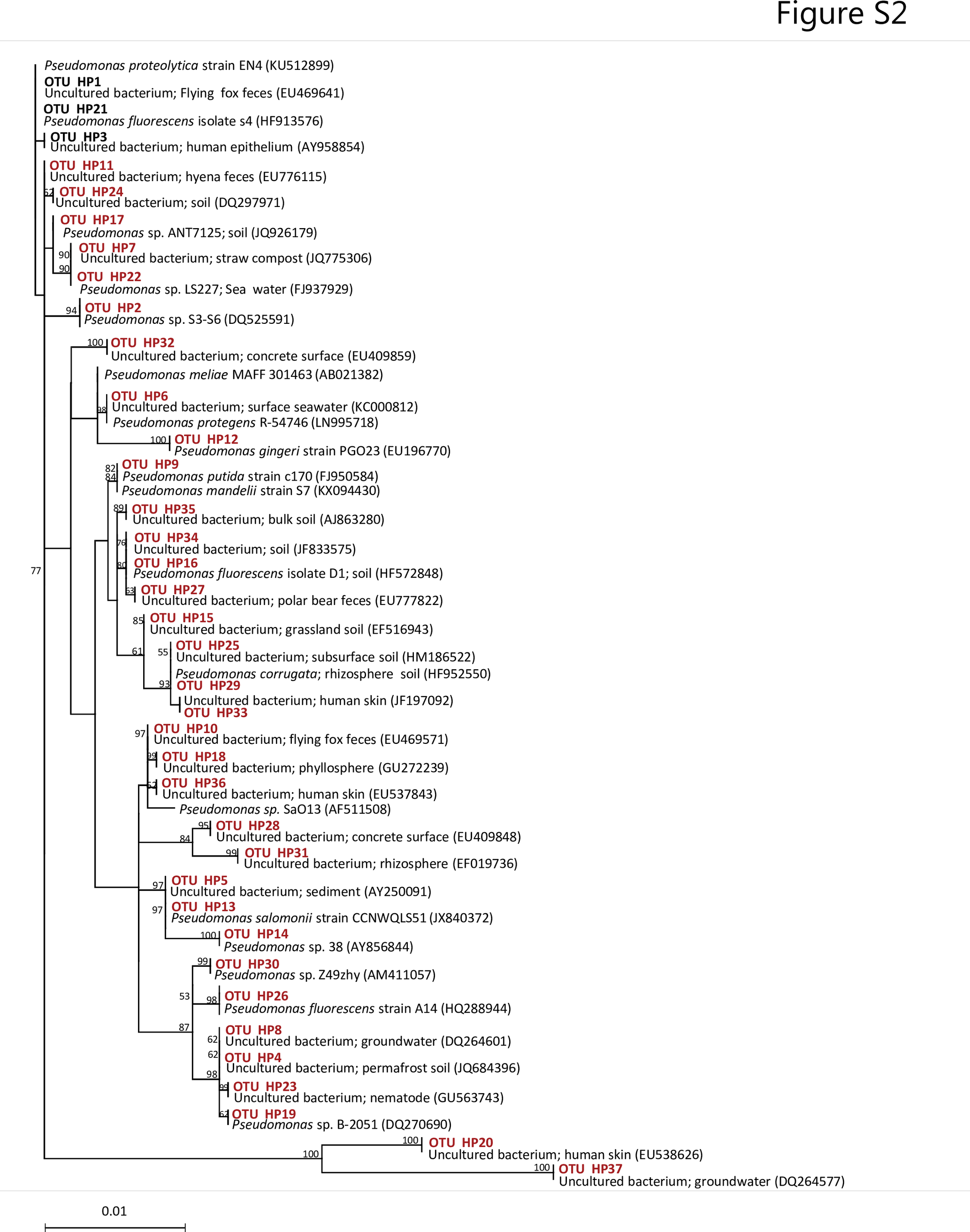

Supplement: S2 Fig — The trees were constructed from 375–400 unambiguously aligned nucleotides from the V3-V4 regions of the 16S rRNA gene. Sequences derived from hyphae-associated bacteria from the microcosms are designated OTU HP/HM. Bootstrap values are based on 1000 pseudoreplications, and only values > 50% are shown. (TIFF) [file pone.0187116.s002.tiff]

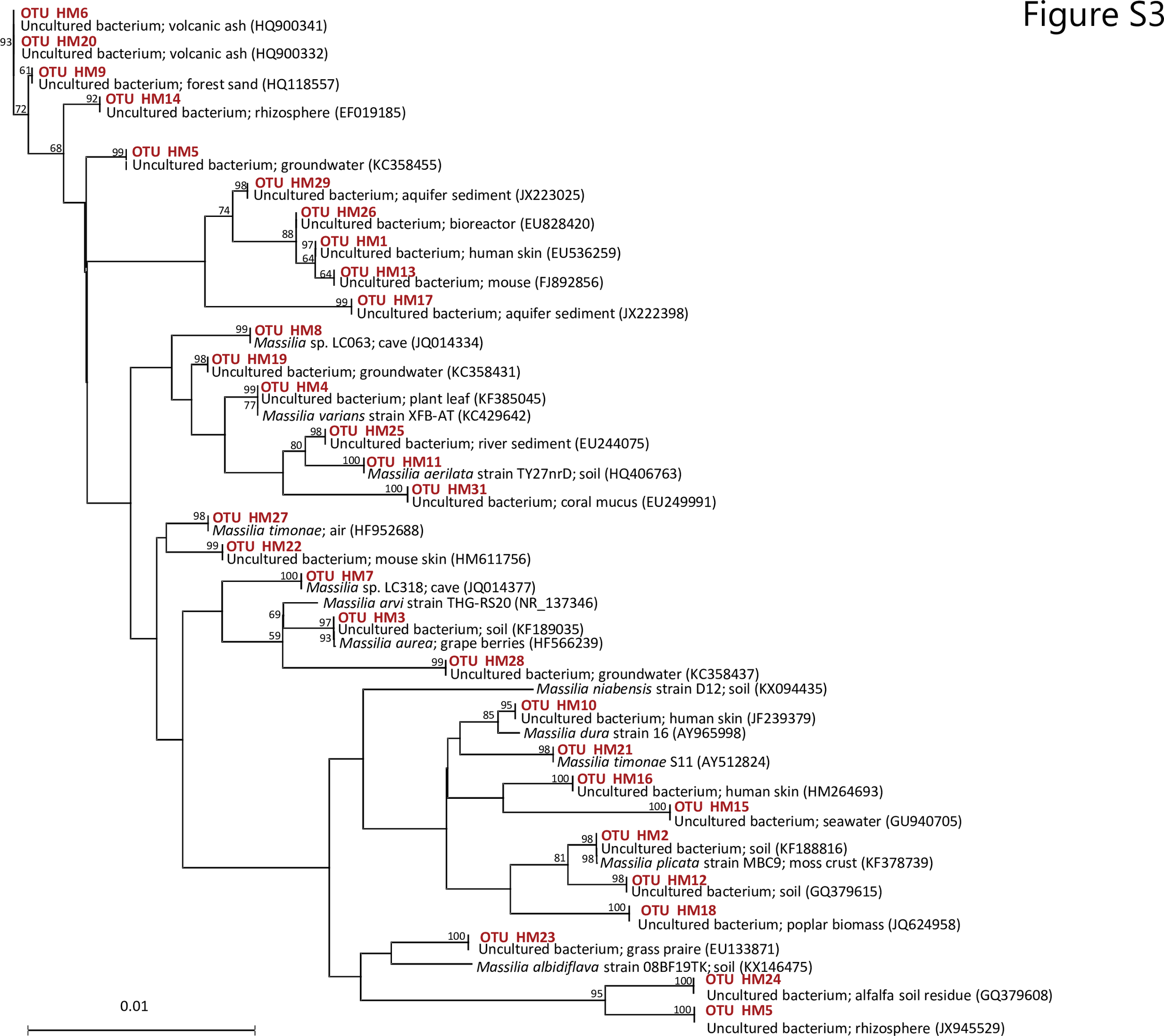

Supplement: S3 Fig — The trees were constructed from 375–400 unambiguously aligned nucleotides from the V3-V4 regions of the 16S rRNA gene. Sequences derived from hyphae-associated bacteria from the microcosms are designated OTU HP/HM. Bootstrap values are based on 1000 pseudoreplications, and only values > 50% are shown. (TIFF) [file pone.0187116.s003.tiff]
